# Alzheimer's Disease Frontal Cortex Single Nuclei Transcriptomic Analysis Reveals Allelic and Ancestral Variation in *APOE* Expression

**DOI:** 10.1002/alz70855_103564

**Published:** 2025-12-24

**Authors:** Sofia Moura, Katrina Celis, Luciana Bertholim Nasciben, Farid Rajabli, Kara L Hamilton‐Nelson, Patrice G Whitehead, Marla Gearing, David A. A. Bennett, Sandra Weintraub, Changiz Geula, William K. Scott, David A. Davis, Regina T Vontell, Theresa Schuck, Derek M. Dykxhoorn, Margaret Pericak‐Vance, Anthony J Griswold, Juan I Young, Jeffery M Vance

**Affiliations:** ^1^ University of Miami Miller School of Medicine, Miami, FL, USA; ^2^ John P. Hussman Institute for Human Genomics, University of Miami Miller School of Medicine, Miami, FL, USA; ^3^ Emory University School of Medicine, Atlanta, GA, USA; ^4^ Department of Neurological Sciences, Rush University Medical Center, Chicago, IL, USA; ^5^ Northwestern ADC Neuropathology Core, Northwestern University Feinberg School of Medicine, Chicago, IL, USA; ^6^ Department of Pathology and Laboratory Medicine, Institute on Aging and Center for Neurodegenerative Disease Research, The Perelman School of Medicine at the University of Pennsylvania, Philadelphia, PA, USA; ^7^ John P. Hussman Institute for Human Genomics, Miller School of Medicine, Miami, FL, USA; ^8^ John P. Hussman Institute for Human Genomics, Miller School of Medicine, University of Miami, Miami, FL, USA

## Abstract

**Background:**

*APOE4* is the strongest genetic risk factor for Alzheimer's disease, with differing risk across populations ‐ European ancestry has higher risk than African ancestry. The local ancestry (LA) surrounding the *APOE4* region was shown to primarily affect this risk difference, with European LA (ELA) carriers having greater *APOE4* chromatin accessibility and expression than African LA (ALA) individuals. Here, we investigated whether *APOE* LA associates with differential expression in *APOE3* allele carriers, potentially revealing ancestry‐specific *APOE* expression regulatory mechanisms.

**Method:**

We performed single nuclei RNA‐seq on frontal cortex (BA9) from individuals with AD, specifically 4 ELA and 4 ALA, all homozygous for *APOE3* and LA. Differential expression and pathways analyses were performed using Seurat, MAST, and gProfiler.

**Result:**

We integrated our *APOE3* data with our group's published data on *APOE4* carriers of the same ancestries. We analyzed 145,551 nuclei from 25 brains (14 ELA and 11 ALA) and identified 35 cell clusters with similar cell proportions/cluster between ancestries. Contrary to our previous findings in *APOE4* carriers where ELA carriers expressed significantly greater *APOE4* than ALA carriers, we observed ALA *APOE3* carriers express higher *APOE* in astrocytes and microglia than ELA carriers. Further, within the same LA, we observed greater *APOE* expression in *APOE4* carriers than *APOE3* carriers. Interestingly, we also observed significant up‐ and down‐regulation of key AD‐related pathways such as lipid response, neurogenesis, phagocytosis, nervous system development, immune response, insulin resistance, oxidative phosphorylation, among others.

**Conclusion:**

Our data suggest *APOE* LA regulates *APOE* expression in an allele‐specific manner. It remains unclear whether this effect is driven by the *APOE* allele itself or differing regulatory elements in the allele haplotypes. Overall, we observed greater *APOE* expression in *APOE4* carriers than *APOE3* carriers across cell types. Given higher *APOE4* expression is associated with increased risk, lower *APOE3* expression may contribute to lower risk. Altogether, this provides additional insights into the expression regulatory mechanisms in the different *APOE* alleles, aiding in therapeutic efforts.

